# InfAcrOnt: calculating cross-ontology term similarities using information flow by a random walk

**DOI:** 10.1186/s12864-017-4338-6

**Published:** 2018-01-19

**Authors:** Liang Cheng, Yue Jiang, Hong Ju, Jie Sun, Jiajie Peng, Meng Zhou, Yang Hu

**Affiliations:** 1 College of Bioinformatics Science and Technology, Harbin Medical University, Harbin, 150081 People’s Republic of China; 2 Hospital for Sick Children, Toronto, M5G 1X8 Canada; 3 Department of Information Engineering, Heilongjiang Biological Science and Technology Career Academy, Harbin, 150081 People’s Republic of China; 4 School of Computer Science, Northwestern Polytechnical University, Xian, 710072 People’s Republic of China; 5 School of Life Science and Technology, Harbin Institute of Technology, Harbin, 150088 People’s Republic of China

**Keywords:** Biomedical ontology, Term similarities, Random walk, Information flow

## Abstract

**Background:**

Since the establishment of the first biomedical ontology Gene Ontology (GO), the number of biomedical ontology has increased dramatically. Nowadays over 300 ontologies have been built including extensively used Disease Ontology (DO) and Human Phenotype Ontology (HPO). Because of the advantage of identifying novel relationships between terms, calculating similarity between ontology terms is one of the major tasks in this research area. Though similarities between terms within each ontology have been studied with in silico methods, term similarities across different ontologies were not investigated as deeply. The latest method took advantage of gene functional interaction network (GFIN) to explore such inter-ontology similarities of terms. However, it only used gene interactions and failed to make full use of the connectivity among gene nodes of the network. In addition, all existent methods are particularly designed for GO and their performances on the extended ontology community remain unknown.

**Results:**

We proposed a method InfAcrOnt to infer similarities between terms across ontologies utilizing the entire GFIN. InfAcrOnt builds a term-gene-gene network which comprised ontology annotations and GFIN, and acquires similarities between terms across ontologies through modeling the information flow within the network by random walk. In our benchmark experiments on sub-ontologies of GO, InfAcrOnt achieves a high average area under the receiver operating characteristic curve (AUC) (0.9322 and 0.9309) and low standard deviations (1.8746e-6 and 3.0977e-6) in both human and yeast benchmark datasets exhibiting superior performance. Meanwhile, comparisons of InfAcrOnt results and prior knowledge on pair-wise DO-HPO terms and pair-wise DO-GO terms show high correlations.

**Conclusions:**

The experiment results show that InfAcrOnt significantly improves the performance of inferring similarities between terms across ontologies in benchmark set.

**Electronic supplementary material:**

The online version of this article (10.1186/s12864-017-4338-6) contains supplementary material, which is available to authorized users.

## Background

Bio-ontology has drawn more and more attention in the standardization of terminology [1–3], functional annotation of molecules and so on [4–7]. Especially, the relationships between terms of an ontology play an important role in clustering gene expression data for yielding biologically meaningful gene clusters [8], prioritizing disease genes for predicting novel disease-causing genes and etc. [9–11].

Nowadays, over 300 biomedical ontologies have been manually curated [12, 13]. These ontologies are established for describing different types of characteristics of molecules, such as participation in biological processes (BP), induction of diseases, and so on. As the wide application of relationships in single ontology, relationships between terms across ontologies would significantly increase interoperability between molecules in multiple aspects and enable new intelligent bioinformatics applications [14].

**Fig. 1:**
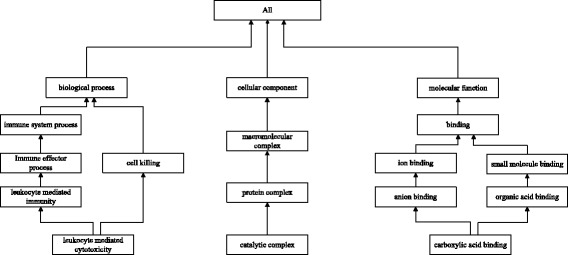
Sub-graph of the Directed Acyclic Graph of three GO sub-ontologies. Each node indicates a term of GO, and each arrow symbol represents an ‘IS_A’ relationship of GO. For example, “catalytic complex” is linked to “protein complex” by an ‘IS_A’ relationship

Gene Ontology (GO) is the earliest and most frequently used ontology, which contains three sub-ontologies (categories) describing molecular function (MF), BP and cellular component (CC) of genes and gene products (Fig. 1). Intra-relationships between terms of each sub-ontology have been manually curated [15] and quantitatively measured [16–19] for dozens of years. By contrast, less attention has been paid to inter-relationships between terms of the tree sub-ontologies. Although several methods have been developed to calculate similarities between terms across these sub-ontologies [20–22], it remains a challenge to achieve high reliability.

Since GO has been widely utilized to annotate genes and gene products of various organisms [5], relationships between its terms can also be reflected by their annotated genes. Accordingly, three state-of-art algorithms were designed to identify term relationships among the three sub-ontologies, which include Association Rule Mining (ASR) method [20], Vector Space Model (VSM) method [21], and Cross-Category Gene Ontology Measurement (CroGO) method [22, 23]. ASR method was initially designed to identify products frequently bought together [24]. It was introduced to calculate similarity between terms across sub-ontologies based on the frequency of their annotated gene sets [20]. Subsequently, inter-relationships identified by ASR method across GO’s three sub-ontologies were integrated into GO as a complement [25]. VSM method describes each GO term as a vector of genes based on a given annotation database [21]. Then the relationships between terms can be measured by the cosine of their corresponding vectors. Both ASR and VSM methods assume genes are independent and ignore the functional interactions between genes which actually contain valuable information about their corresponding terms. Gene functional interaction network (GFIN) is the widely accepted source of gene interactions at present [26–29]. CroGO utilizes GFIN to enhance its power for the calculation of similarity between terms [22]. It benefits from the additional information stored within gene interaction network which implicates correlations of genes’ annotation terms. However, CroGO calculates the similarity between terms only through considering interactions between their annotated genes, but ignores the connectivity among gene nodes of the network. All of these three methods were designed and validated for measuring similarities between terms across GO’s three sub-ontologies. They should have the potential to be applied on ontologies built after GO such as Disease Ontology (DO) (Kibbe et al. 2015) and Human Phenotype Ontology (HPO) [30]. However, little work has been done on this aspect.

In this study, we proposed a new method InfAcrOnt to calculate similarities between terms across ontologies utilizing the entire GFIN. In our model, a weighted term-gene-gene network (WTGGN) is created by combining gene annotations and GFIN. Then the information flow in the network is modeled by a random walk [31, 32] to calculate term similarities. The method has been validated with experiments on multiple ontologies including DO and HPO.

## Methods

**Fig. 2:**
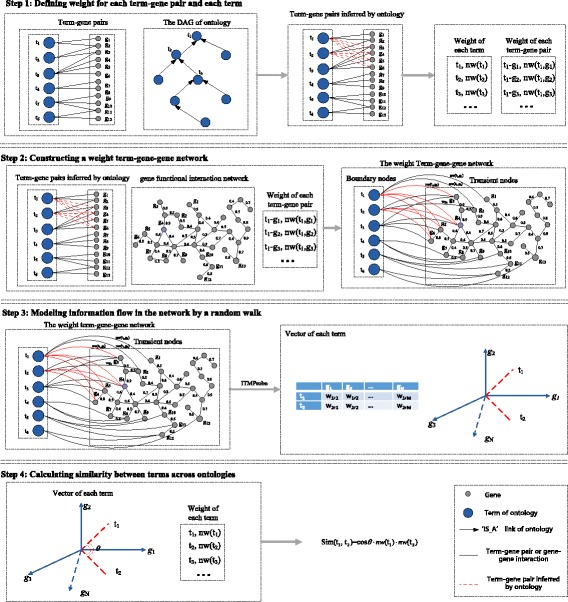
Overview of InfAcrOnt demonstrating the basic ideas of measuring similarity between terms across ontologies

InfAcrOnt has four steps to measure similarities between terms across different ontologies (Fig. 2). First, the weight of term-gene pair was defined. Each of the term-gene pairs was got from a functional annotation of gene. We also define weight of each term in the ontologies. Second, we built a WTGGN based on the weighted term-gene pairs and weighted gene interactions from GFIN. Third, each term was represented as a vector of genes through modeling information flow in the WTGGN by random walk. The dimension of vector equals to the number of genes in the network. Fourth, we calculated cosine between vectors and adjust the value with the weight of terms. The results are used as similarities between terms.

### Step 1: Defining weight for each term-gene pair and each term

Ontology annotations provide functional annotations for each gene. Each entry of annotations can be extracted as a term-gene pair. To construct a WTGGN, we define the weight of term-gene pair according to the importance of the gene to a term-gene pair in Eq. 1, which is inversely proportional to the total number of terms related to gene. Assuming a gene is annotated with only one term, this term-gene relationship should be very important for the WTGGN. On the contrary, if a gene is annotated with multiple terms, the importance of each of these term-gene relationships should be divided equally.

1 $$ \mathrm{w}\left({\mathrm{t}}_{\mathrm{i}},{\mathrm{g}}_j\right)=- lo{g}_2\frac{n_j}{N_T} $$

where *n*_*j*_ represents the number of terms associated with the gene *g*_*j*_, *N*_*T*_ represents the number of all the annotation terms. Then the weight of each term-gene pair is normalized with Eq. 2.

2 $$ \mathrm{nw}\left({\mathrm{t}}_{\mathrm{i}},{\mathrm{g}}_{\mathrm{j}}\right)=\frac{w\left({\mathrm{t}}_{\mathrm{i}},{\mathrm{g}}_{\mathrm{j}}\right)-{wtg}_{\mathrm{min}}}{wtg_{\mathrm{max}}-{wtg}_{\mathrm{min}}}, $$

where *wtg*_*min*_ and *wtg*_*max*_ are the minimum and maximum weights of term-gene pairs, respectively.

In an ontology, terms are stored as nodes in a Directed Acyclic Graph (DAG) which are connected with ‘IS_A’ relationship (Fig. 1). According to the set inclusion relation by ‘IS_A’ relationships [33], if a gene is annotated by a term, then the gene is also annotated by its ancestors. Therefore, terms in the higher level of the DAG can annotate more genes which lead to shallow annotation [34]. Theoretically, the depth of a term in DAG should be inversely proportional to the number of the genes it annotates. To avoid this problem we assign a weight to each term. The weight of term is defined in Eq. 3 in which terms with fewer annotated genes are assigned to relatively higher weight.

3 $$ \mathrm{w}\left({\mathrm{t}}_{\mathrm{i}}\right)=- lo{g}_2\frac{n_g}{N_G}, $$

where *n*_*g*_ represents the number of genes annotated by term *t*_*i*_, *N*_*G*_ represents the number of all annotated genes. Then the weight of each term is normalized between 0 and 1 with Eq. 4.

4 $$ \mathrm{nw}\left({\mathrm{t}}_{\mathrm{i}}\right)=\frac{w\left({\mathrm{t}}_{\mathrm{i}}\right)-{wt}_{\mathrm{min}}}{wt_{\mathrm{max}}-{wt}_{\mathrm{min}}}, $$

where *wt*_*min*_ and *wt*_*max*_ are the minimum and maximum weights of terms, respectively.

### Step 2: Constructing a weighted term-gene-gene network

We then construct a WTGGN with weighted term-gene pairs achieved in step1 and weighted gene-gene interaction in GFIN. In this network, there are two types of nodes, term nodes and gene nodes. There are also two types of edges, term-gene edge (term node to gene node) and gene-gene edge (gene node to gene node). Each term-gene edge weight is calculated with Eqs. 1 & 2 and gene-gene weight is sourced from GFIN. The latter is further normalized with Eq. 5.

5 $$ \mathrm{w}\left({\mathrm{g}}_{\mathrm{i}},{\mathrm{g}}_{\mathrm{j}}\right)=\frac{FIS\left({\mathrm{g}}_{\mathrm{i}},{\mathrm{g}}_{\mathrm{j}}\right)-{FIS}_{\mathrm{min}}}{FIS_{\mathrm{max}}-{FIS}_{\mathrm{min}}}, $$

where *FIS(g*_*i*_*, g*_*j*_*)* represents functional interaction score between genes *g*_*i*_ and *g*_*j*_ from GFIN, *FIS*_*min*_ and *FIS*_*max*_ are the minimum and maximum weights of gene-gene edges, respectively.

The WTGGN contains all necessary information for the calculation of similarities between terms across ontology. This information involves term-gene pairs of ontology annotations and gene-gene interactions of GFIN. In other words, term nodes can be connected by interactions between their annotated gene nodes and intermediate gene nodes in the network, which provides a potential possibility to calculate term similarity more comprehensively.

### Step 3: Modeling information flow in the network by a random walk

Three models have been designed for modeling information flow by a random walk with damping in the network, such as absorbing, emitting and channel models [35, 36]. The random walk starts from source nodes and terminates either by dissipation or by reaching a sink node. Source nodes and sink nodes are boundary nodes while others are transient nodes. Unlike the classical random walk, these models allow the walker to dissipate or damp at each step under a certain probability. Each walk, if not dissipated, simulates a possible information path from source node to sink node. Absorbing model assigns nodes the random walk ends at, emitting model assigns nodes the random walk starts from and channel model integrates both absorbing and emitting models for directed information flow.

Information Transduction Module (ITM) Probe [37] program has implemented all of these three models. It outputs the expected number of visits to each transient and sink node by random walker originated from every node. The ITM takes an undirected network as input, for each source node it searches for a path to sink nodes under a given dissipation rate. Smaller dissipation rate allows random walks to explore nodes farther to the source while larger dissipation rate evaporates most walks more quickly. In channel model, dissipation rate controls how much a random walk can deviate from the shortest path from sources to sinks. The expected number of visits from the transient nodes to source nodes in the network are scored and returned in terms of the weights by ITM Probe.

Channel model is applied on our WTGGN by ITM Probe. All genes in the network are transient nodes. To access the weight of each gene for a given term, we specified the term as the source node and sink node based on the network. Based on this method, a term could be represented as a weighted vector. Each dimension of the vector is the weight score of a gene to the term. Through random walk in the channel model, the connectivity of the entire network of GFIN can be fully utilized.

Here, the damping factor equals 0.85 according to the previous study [35]. Assuming *N* genes exist in the WTGGN, each term can be represented as N-dimension vector based on channel model through the ITM Probe. For a given term *t*_*1*_, the weighted vector can be described as:

6 $$ {WV}_{{\mathrm{t}}_{\mathrm{i}}}=\left\{{w}_{i,1},{w}_{i,2},\dots, {w}_{i,N}\right\}, $$

where *WV*_*ti*_ means a weighted vector of *t*_*i*_, and *w*_*i,j*_ represents the weight score of *t*_*i*_ on the *j*th dimension.

### Step 4: Calculating similarities between terms across ontologies

Then we define the similarity between term *t*_*1*_ and *t*_*2*_ as following:

7 $$ Sim\left({t}_{\mathrm{i}},{t}_j\right)=\cos \left(\theta \right)\cdot nw\left({t}_i\right)\cdot nw\left({t}_j\right), $$

8 $$ \cos \left(\theta \right)=\frac{\sum \limits_{n=1}^N{w}_{i,n}\cdot {w}_{j,n}}{\sqrt{\sum \limits_{n=1}^N{w_{i,n}}^2}\sqrt{\sum \limits_{n=1}^N{w_{j,n}}^2}}, $$

where the cosine of the vectors of *t*_*i*_ and *t*_*j*_ is the similarity between terms. The vectors of terms were obtained based on step 3. *nw(t*_*i*_*)* and *nw(t*_*j*_*)* represent the normalized weight of term *t*_*i*_ and *t*_*j*_, which could be calculated based on eqs. 3 & 4. Here, *nw(t*_*i*_*)* and *nw(t*_*j*_*)* is used to avoid shallow annotation. The corresponding algorithm was described in the Additional file 1.

## Results

### Performance evaluation of calculating similarities of pair-wise BP-MF terms

A benchmark set for human has been built by extracting similar pair-wise BP-MF terms in a previous study [22]. Taken pairs of the benchmark set as our positive group (PG), we get random pairs as a negative group (NG). Then the similarity score of PG and NG was calculated to evaluate the performance of existing methods. e.g. The performance of InfAcrOnt should be superior if the similarity score of the PG can be prioritized at the top.

Pair-wise terms of the benchmark set were generated based on their co-occurrence enzymes [25]. On the one hand, BP terms are also defined as the name of metabolic pathways, each of which is associated with several enzymes. On the other hand, MF terms can also be linked to enzymes with the official GO translations [38, 39]. As a result, 80 pairs of BP-MF terms associated with common enzymes based on HumanCyc [40] were obtained for human as PG. Then 10 times (800 pairs) of benchmark set were obtained randomly as a NG. Here each term of random pairs is selected from the terms with annotated genes.

To calculate similarity of term pairs of PG and NG, we need to construct a WTGGN for BP-MF terms and their annotated genes. GO [15] was downloaded from open source repositories (Table 1) which provided manually curated ‘IS_A’ relationships between terms [33]. Currently, a total of 12,174 ‘IS_A’ relationships between 9988 MF terms and 54,502 ‘IS_A’ relationships between 28,245 BP terms are included in these ontologies. GO annotations (GOA) of human genes were accessed from GO Consortium (Table 1). Each entry of annotation of GOA was tagged with a GO evidence code. An annotation with the evidence code ‘IEA’ means it is non-experimental annotation without confirmed by a human annotator. After removing ‘IEA’ annotations, 3217 MF terms and 9032 BP terms are used to annotate 14,435 human genes which generate 132,984 annotations were obtained. To the best of our knowledge, HumanNet [29] is the latest and most frequently used GFINs for human. Currently, HumanNet contains 476,399 interactions among 16,243 human genes. Using GO, GOA and HumanNet a WTGGN for BP-MF terms and their annotated genes was constructed based on step 1 and step 2 of the ‘Methods’ section. Then the similarity of term pairs of PG and NG was calculated based on step 3 and step 4 of the ‘Methods’ section.

**Table 1:** Data sources used for identifying novel relationships across ontologies

Data source	Web site
GO	http://geneontology.org/page/download-ontology
GOA for yeast	http://geneontology.org/gene-associations/gene_association.sgd.gz
GOA for human	http://geneontology.org/gene-associations/gene_association.goa_ref_human.gz
YeastNet	http://www.inetbio.org/yeastnet/
HumanNet	http://www.functionalnet.org/humannet/
HPO & HPOA	http://human-phenotype-ontology.github.io/
DO	http://disease-ontology.org/
DOA	http://www.bio-annotation.cn/gene2function/
PubMedA	http://www.bio-annotation.cn/ARSSIC

**Fig. 3:**
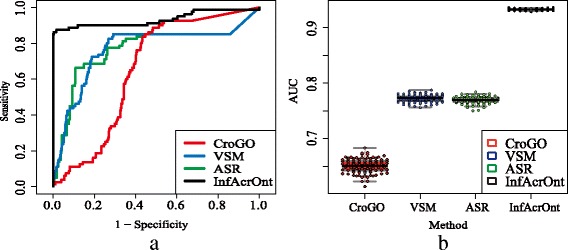
ROC analysis of the benchmark set and random sets for human. **a** ROC curves for the experimental results on the benchmark set and a random set for human. It shows 1-specificity versus sensitivity of each method for calculating the similarities of terms across BP and MF. **b** Average of AUC for 100 iterators for human

The performances of existing methods are assessed by drawing a receiver operating characteristic (ROC) curve. We can get true-positive (TP), false-positive (FP), true-negative (TN), and false-negative (FN) using various similarity scores of PG and NG as threshold. Then the curve is created by plotting the true positive rate (TPR = (TP) / (TP + FN)) (or Sensitivity) against the false positive rate (FPR = 1-(TN) / (TN + FP)) (or 1-Specificity) at various threshold settings. The area under the ROC curve (AUC) showed the performance of each method for distinguishing PG from NG. Figure 3a shows a ROC curve of the existing method based on our PG and a NG. The corresponding AUCs by the CroGO, VSM, ASR, and InfAcrOnt methods are 0.6539, 0.7674, 0.7659, and 0.9330 respectively. ASR and VSM methods are the two classical approaches. The similar ROCs of these two methods show that the performances of these two methods are almost the same. Although CroGO method introduced the interactions between genes, it did not perform well. This may be caused by the fact that the connectivity between genes through the GFIN wasn’t be utilized. Fortunately, the entire GFIN was incorporated in the InfAcrOnt method. And the significantly higher AUC (0.9330) validates that our method helps to enhance the true positive rate and reduces the false positive rate.

The experiment was iterated 100 times based on 100 random NGs. The AUCs of 100 iterations are shown in Fig. 3b. The average AUCs of the CroGO, VSM, ASR, and InfAcrOnt methods are 0.6509, 0.7721, 0.7690, and 0.9322 respectively. And the corresponding standard deviations (SDs) of these AUCs are 1.5699e-4, 3.9732e-5, 3.5278e-5, and 1.8746e-6 respectively. In comparison with other methods, AUC is improved more than 0.15 by InfAcrOnt. The highest AUC and lowest SD of InfAcrOnt shows a significant advantage of our method.

Peng et al. also provide another benchmark set of similar pair-wise BP-MF terms for yeast [22]. Then the similar experiment was done on yeast. The set was generated based on their co-occurrence enzymes [25]. Finally, 175 pairs of BP-MF terms related with common enzymes by YeastCyc [41] were obtained for yeast as PG, and corresponding 1750 random pairs were obtained as a NG. The WTGGN for calculating term pair similarity was built based on GO, GOA for yeast, and YeastNet [28]. After removing ‘IEA’ annotations, 1676 MF terms and 2655 BP terms are used to annotate 6332 yeast genes which generate 26,488 annotations were obtained. YeastNet [28] is the latest and most frequently used GFINs for yeast. It includes 362,421 interactions between 5809 yeast genes.

The results of benchmark set for yeast are shown in Additional file 2. According to this figure, the AUCs of one of our experiments for yeast by the CroGO, VSM, ASR, and InfAcrOnt methods are 0.6689, 0.7640, 0.7660, and 0.9307 respectively. The AUCs of 100 iterations for yeast are shown in Additional file 2. The average AUCs of the CroGO, VSM, ASR, and InfAcrOnt methods for yeast are 0.6546, 0.7608, 0.7664, and 0.9308 respectively. And the corresponding SDs of these AUCs are 4.3988e-5, 2.1204e-5, 1.6300e-5 and 3.0977e-6 respectively. These results show the consistency in both human and yeast. This indicates that the advantage of InfAcrOnt in calculating similarity of BP-MF terms is stable and reliable.

### Performance evaluation of calculating similarities of pair-wise DO-HPO terms

To show InfAcrOnt’s ability to work on ontologies other than GO’s 3 sub-ontologies, we calculated similarities of pair-wise DO-HPO terms. The similarity of DO-HPO term pairs can also be calculated based on prior knowledge in HPO project [42] by Term Frequency Inverse Document Frequency (TF-IDF) [43]. Theoretically, similarity score between terms based on genes should be consistent with this based on phenotypes. Therefore, we calculated the Pearson correlation coefficient between InfAcrOnt similarity score and TF-IDF similarity score to evaluate the performance of InfAcrOnt.

A WTGGN for DO-HPO terms and their annotated genes was built by DO, HPO, DO Annotations (DOA), HPO Annotations (HPOA), and HumanNet (Table 1). Then the similarities of pair-wise DO-HPO terms were calculated by InfAcrOnt based on the WTGGN. DO [44] and HPO [30] were downloaded from open source repositories (Table 1) which provided manually curated ‘IS_A’ relationships between terms [33]. Currently, 15,459 ‘IS_A’ relationships between 11,673 HPO terms and 7124 ‘IS_A’ relationships of 6920 DO terms are included in these ontologies. DOA [10] were sourced from the annotations of GeneRIF [45]. After removing duplication, 98,008 associations between 2576 diseases and 9991 genes were obtained. HPOA of human genes were accessed from the HPO project [42] which provided annotated genes relative to human phenotype. Currently, it contains 120,890 associations between 5838 terms and 3496 genes. HumanNet has been accessed in 3.1 section.

HPO project [42] parsed textual descriptions of each disease in the Clinical Synopsis section of OMIM entry. And the phenotypes of the textual descriptions were extracted and organized into HPO. Diseases of OMIM entries were mapped to DO terms based on cross-reference [44, 46]. Notably, a phenotype occurred in textual descriptions of a disease only shows a text relevance between the phenotype and the disease. Thus we need to quantify this text relevance. To this end, we constructed a n-by-m matrix where N was the number of DO terms and M was the number of HPO terms. The (*i*th, *j*th) element of the matrix was valued with the number of occurrences of *j*th row phenotype in the textual descriptions of *i*th disease. Subsequently, we applied TF-IDF [43], a typical model for quantifying text relevance, to calculate the similarity between HPO terms and DO terms based on the matrix.

**Fig. 4:**
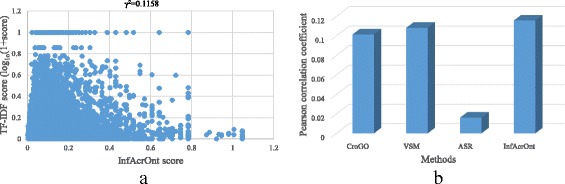
The correlation between the term similarity based on ontology annotations and prior knowledge in HPO project. **a** The distribution of the similarity scores by InfAcrOnt method. **b** Pearson Correlation Coefficient between similarity scores based on TF-IDF and other methods

Figure 4 shows the correlation between InfAcrOnt similarity score and TF-IDF similarity score (Pearson correlation, γ^2^ = 0.1158 *p* = 2.2e-16). The high correlation validated the good performance of InfAcrOnt in calculating the similarity of DO-HPO terms. To further test the performance of the proposed method, InfAcrOnt was compared with the state-of-art methods including ASR, VSM, and CroGO. The comparison results are shown in Fig. 4b and Additional file 3. The similarity based on the ARS method accessed the lowest correlation with the TF-IDF similarity (Pearson correlation, γ^2^ = 0.0163 *p* = 0.0062), which is shown in Fig. 4b and Additional file 3 In comparison, the TF-IDF similarity is more correlated with the similarity based on the CroGO method (Pearson correlation, γ^2^ = 0.1015 p = 2.2e-16; Fig. 4b and Additional file 3), the VSM method (Pearson correlation, γ^2^ = 0.1083 p = 2.2e-16; Fig. 4b and Additional file 3). As expected, similar terms could be identified based on existing methods and prior knowledge in HPO project simultaneously. In comparison with these state-of-art methods, InfAcrOnt similarity achieves the most correlation with prior knowledge.

### Performance evaluation of calculating similarities of pair-wise DO-BP terms

To show InfAcrOnt’s ability to calculate similarity between terms across GO’s sub-ontologies and other ontologies, we applied our method in DO-BP terms. An alternative way to calculate the similarity of DO-BP term pairs is based on prior knowledge in PubMed [47] by the Extensional Mutual Information (EMI) method [48]. Then the correlation between InfAcrOnt similarity score and EMI similarity score was utilized to evaluate the performance of InfAcrOnt.

A WTGGN for DO-GO terms and their annotated genes was constructed by DO, GO, DOA, GOA and HumanNet. All of these data have been accessed in 3.1 and 3.2 sections. Then the similarities of pair-wise DO-BP terms were calculated by InfAcrOnt based on the WTGGN.

Literature of PubMed documents DO terms and GO terms in its title and abstract. Two terms occur in a literature is defined as a co-occurrence relationship between them [48]. These co-occurrence relationships can be quantified as the similarity of DO-BP term pairs. One of the most frequently used algorithm to do this is EMI by Wren et al. [48]. Here we downloaded the co-occurrence relationships of DO-BP term pairs in PubMed from the previous study [9], and then calculated the EMI similarity of DO-BP term pairs.

**Fig. 5:**
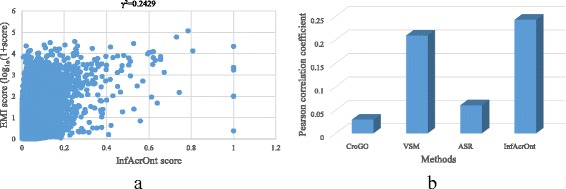
The correlation between the term similarity based on ontology annotations and prior knowledge in PubMed. **a** The distribution of the similarity scores by InfAcrOnt method. **b** Pearson Correlation Coefficient between similarity score based on EMI and other methods

Figure 5 shows the correlation between InfAcrOnt similarity score and EMI similarity score (Pearson correlation, γ^2^ = 0.2429 *p* = 2.2e-16). The high correlation validated the good performance of InfAcrOnt in calculating the similarity of DO-BP terms. To further test the performance of the proposed method, InfAcrOnt was compared with ASR, VSM, and CroGO. The comparison results are shown in Fig. 5b and Additional file 4. As expected, the results show that EMI similarity is also positive correlated with the similarity based on the CroGO method (Pearson correlation, γ^2^ = 0.0296 p = 2.2e-16; Fig. 5b and Additional file 4), the VSM method (Pearson correlation, γ^2^ = 0.2092 p = 2.2e-16; Fig. 5b and Additional file 4), the ASR method (Pearson correlation, γ^2^ = 0.0605 p = 2.2e-16; Fig. 5b and Additional file 4). In comparison, the similarity based on the InfAcrOnt method is the most relevant with the EMI similarity.

### Case studies: diabetes mellitus, alzheimer’s disease, and neuroblastoma related biological process

To further indicate our method in identifying disease-related BP, case studies of Diabetes Mellitus (DM), Alzheimer’s Disease (AD), and Neuroblastoma were examined. The similarity of DO-BP terms was calculated in section 3.3. Here we ranked the BP terms of diseases by the InfAcrOnt similarity score, and then investigated top 5 similar BP terms of these three diseases respectively. Their relationships were manually checked in the published studies and the results were listed in Additional file 5. All of five DM-BP relationships were validated. And four of five AD-BP relationships and four of five neuroblastoma-BP relationships were also validated. For example, DM increases reactive oxygen species (GO:0000302) production [49], DNA replication (GO:0006275) stress is a key element of AD [50]. All of these results indicate that our method can function in identifying potential DO-BP terms.

## Discussion

The importance of the relationship between terms across ontologies had been reflected in the previous researches [14, 51, 52]. However, few of these relationships were manually curated in the existing vocabularies. Currently, methods have been developed for measuring the similarity between terms across ontologies based on term-gene pairs of ontology annotations, which can prioritize these inter-relationships [20–22]. Because of ignoring the connectivity of the GFIN, existing methods were limited for identifying novel relationships. To solve this problem, in this article we devised a new method named InfAcrOnt for improving the performance of calculating the similarity of terms across ontologies by integrating ontology annotations and GFIN through information flow.

The performance of InfAcrOnt was validated very well in calculating similarities of BP-MF term pairs according to the evaluation on two benchmark sets (Fig. 3 and Additional file 2). The two benchmark sets were selected strictly by their common enzymes (see ‘3.1’ section). Therefore, our method is very suitable for identifying strong relationships. Because two benchmark sets are sourced from human and yeast, respectively, and the experiment was iterated 100 times, the stability of our method was also proved very well.

The superior performance of InfAcrOnt was also validated in calculating the similarity of pair-wise DO-HPO terms (Fig. 4) and pair-wise DO-GO terms (Fig. 5). The high correlations between similarity based on existing methods and similarity based on prior knowledge show that the performance of the ASR, VSM, CroGO, and InfAcrOnt methods are also good for other ontologies besides sub-ontologies of GO. Considering the fluctuation of the performance of the ASR and CroGO (Figs. 4b and 5b) methods, the VSM and InfAcrOnt methods perform better. In comparison with other methods, InfAcrOnt achieves the highest correlation, which means it is the most consistent with prior knowledge.

Over 300 ontologies have been developed in the biomedical domain. The lack of relationships between terms across these ontologies limited the interoperability in term level. Fortunately, InfAcrOnt can function in identifying novel relationships based on ontology annotations and GFIN. Because most of the ontologies were used to annotate genes and GFIN has been constructed [28, 29], InfAcrOnt can be used widely for calculating similarities between terms across these ontologies. Furthermore, the case studies validate the method can function in identifying novel relationships.

## Conclusions

In this article, we presented a novel method InfAcrOnt for calculating cross-ontology term similarities using information flow by a random walk. The method mainly focused on taking advantage of the connectivity of the GFIN. To validate its performance, experiments were conducted on InfAcrOnt and state-of-art methods on sub-ontologies of GO and other frequently used ontologies. The highest AUC (0.9322 and 0.9309) and lowest SDs (1.8746e-6 and 3.0977e-6) were achieved for InfAcrOnt in both human and yeast benchmark datasets. And the highest correlation were also obtained between similarity score using InfAcrOnt and prior knowledge for DO-HPO (Pearson correlation, γ^2^ = 0.1158 p = 2.2e-16) and DO-BP (Pearson correlation, γ^2^ = 0.2429 p = 2.2e-16) terms. All of these results exhibited the superiority of our method. In the case study, novel identified BPs of DM and AD using InfAcrOnt were verified in recent literatures. Currently, over 300 ontologies without interoperability in term level have been developed in the biomedical domain. Therefore, it is valuable for using InfAcrOnt to mine novel relationships across ontologies.

## Additional files

Additional file 1: Algorithm for measuring term similarities across ontologies. (PDF 193 kb)

Additional file 2: AUC analysis of the benchmark set and random sets for yeast. (PDF 463 kb)

Additional file 3: The correlation between the term similarity by state-of-art methods and prior knowledge in HPO project. (PDF 145 kb)

Additional file 4: The correlation between the term similarity by state-of-art methods and prior knowledge in PubMed. (PDF 134 kb)

Additional file 5: Disease-related biological process confirmed by literature mining. (PDF 102 kb)

## Abbreviations

AD: Alzheimer’s disease
ASR: Association rule mining
AUC: Area under the receiver operating characteristic curve
BP: Biological processes
CC: Cellular component
CroGO: Cross-category gene ontology measurement
DM: Diabetes mellitus
DO: Disease ontology
EMI: Extensional mutual information
FN: False-negative
FP: False-positive
FPR: False positive rate
GFIN: Gene functional interaction network
GO: Gene ontology
GOA: GO annotations
HPO: Human phenotype ontology
HPOA: HPO annotations
ITM: Information transduction module
MF: Molecular function
NG: Negative group
PG: Positive group
ROC: Receiver operating characteristic
SDs: Standard deviations
TF-IDF: Term frequency inverse document frequency
TN: True-negative
TP: True-positive
TPR: True positive rate
VSM: Vector space model
WTGGN: Weighted term-gene-gene network
