# Supplementary material for: InfAcrOnt: calculating cross-ontology term similarities using information flow by a random walk
Source: BMC Genomics. 2018 Jan 19;19(Suppl 1):919. doi: 10.1186/s12864-017-4338-6 (PMC5780854; doi:10.1186/s12864-017-4338-6)
Supplement: Supplementary file 1 — Algorithm for measuring term similarities across ontologies. (PDF 193 kb) [file 12864_2017_4338_MOESM1_ESM.pdf]

Additional file 1. Algorithm for measuring term similarities across ontologies.

ALGORITHM: Algorithm for measuring term similarities across ontologies.

Input: term set $T_1=\{t_{1,1}, t_{1,2}, \ldots , t_{1,n}\}$, 'IS_A' relationships of T_1, term set $T_2=\{t_{2,1}, t_{2,2}, \ldots , t_{2,m}\}$, 'IS_A' relationships of T_2, gene set $G=\{ g_1, g_2, \ldots , g_k \}$, term-gene pairs set TG $\{(t_1,g_1), (t_1,g_2), \ldots(t_m,g_k)\}$, weights of gene-gene pair $\{wg_{1,2}, wg_{1,3},\ldots, wg_p)\}$.

Output: The similarity of all the term pairs.

For each term-gene pair (t_i, g_j) in TG
  Calculate weights of each pair $(w(t_i, g_j))$ using equation 1.
For each term-gene pair (t_i, g_j) in TG
  Normalize weights of each pair $(nw(t_i, g_j))$ using equation 2.
For each term t_i in T_1 or T_2
  Calculate weights of each pair $(w(t_i))$ using equation 3.
For each term t_i in T_1 or T_2
  Normalize weights of each pair $(nw(t_i))$ using equation 4.
For each gene-gene pair (g_i, g_j) in T_1 or T_2
  Normalize weights of each pair $(w(g_i, g_j))$ using equation 5.
For each term t_i in T_1 or T_2
  Calculate the vector of each term (WV_{t_i}) using ITM probe.
For each t_i in T_1
  For each t_j in T_2
    Calculate the similarity of t_i and t_j $(Sim(t_i, t_j))$ using equation 7&8
    add $Sim(t_i, t_j)$ to $Sim(T_1, T_2)$.
return $Sim(T_1, T_2)$.
