# Supplementary material for: InfAcrOnt: calculating cross-ontology term similarities using information flow by a random walk
Source: BMC Genomics. 2018 Jan 19;19(Suppl 1):919. doi: 10.1186/s12864-017-4338-6 (PMC5780854; doi:10.1186/s12864-017-4338-6)
Supplement: Supplementary file 2 — AUC analysis of the benchmark set and random sets for yeast. (PDF 463 kb) [file 12864_2017_4338_MOESM2_ESM.pdf]

Additional file 2. AUC analysis of the benchmark set and random sets for yeast. A. ROC curves for the experimental results on the benchmark set and a random set for yeast. B. Average of AUC for 100 iterators for yeast.

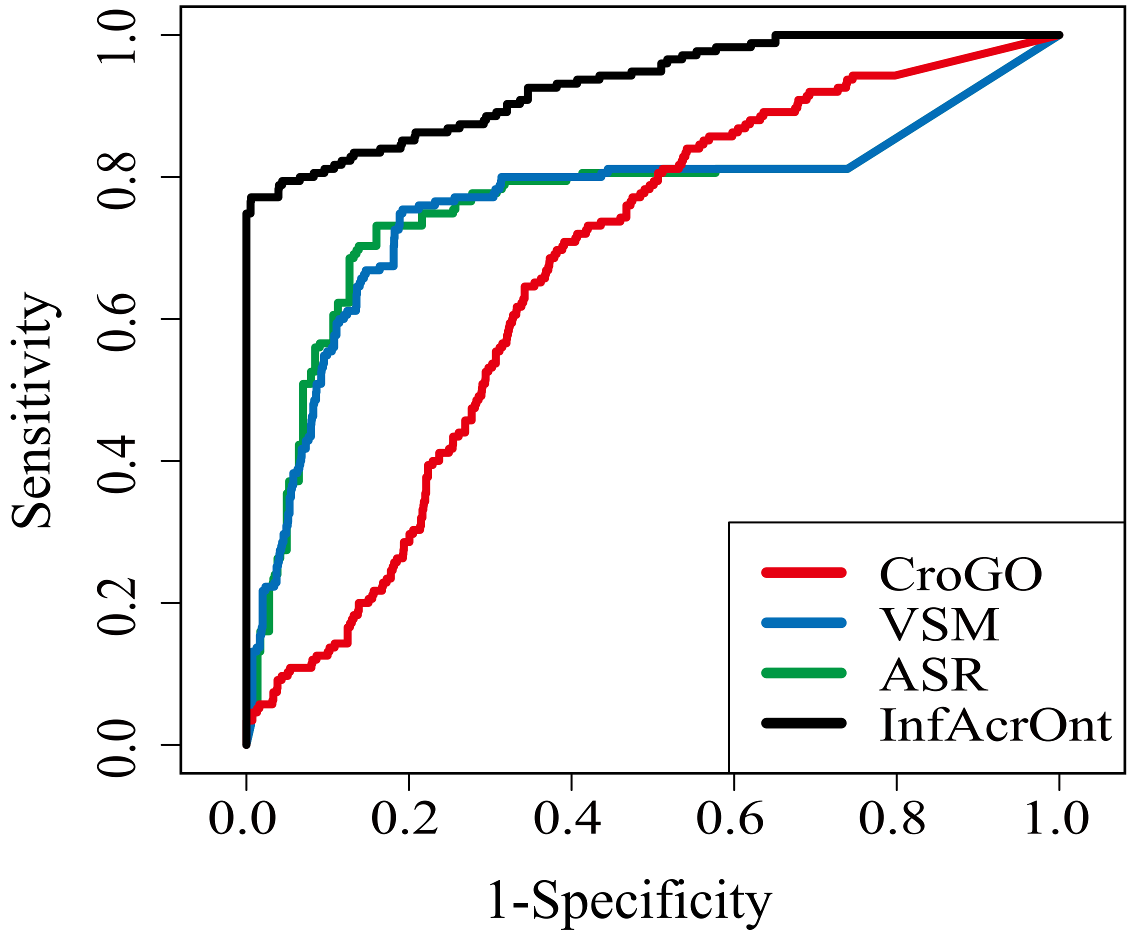

A

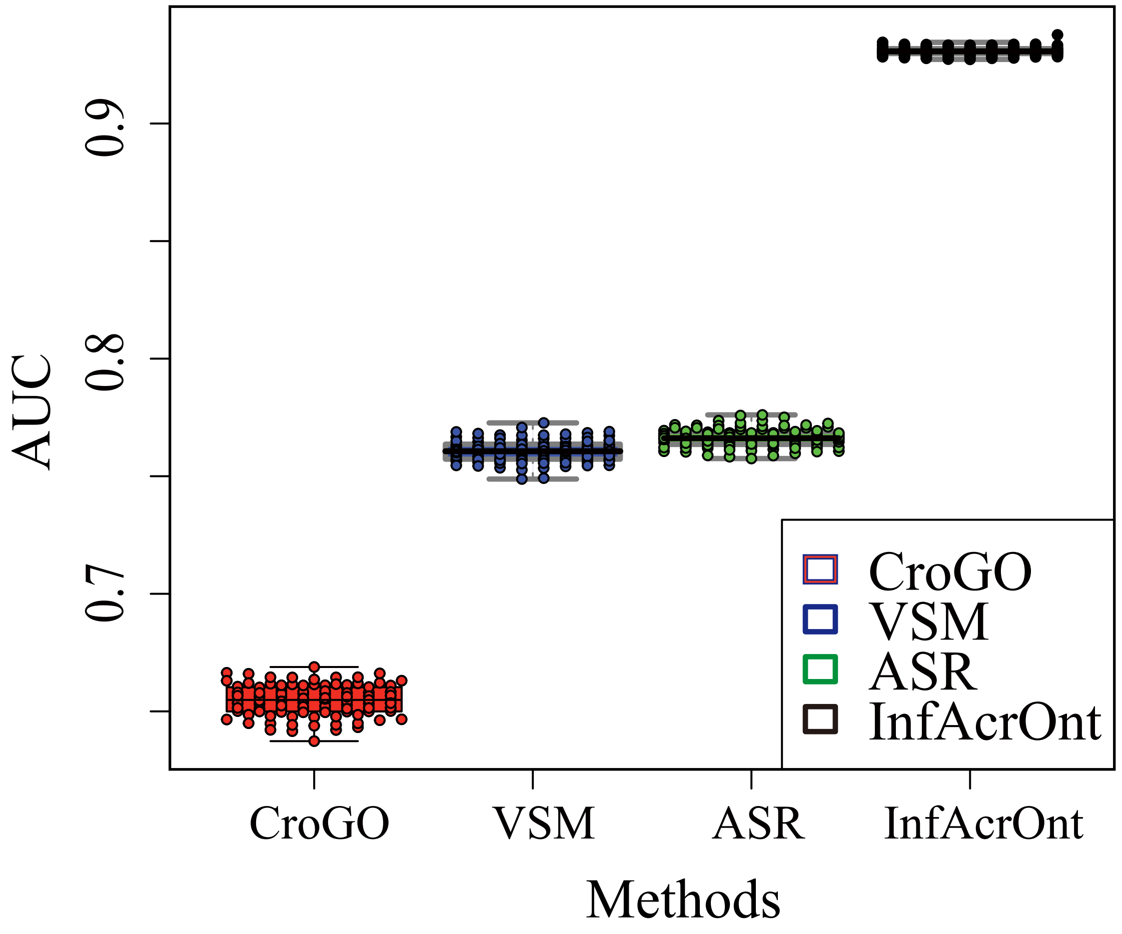

B
