# Supplementary material for: InfAcrOnt: calculating cross-ontology term similarities using information flow by a random walk
Source: BMC Genomics. 2018 Jan 19;19(Suppl 1):919. doi: 10.1186/s12864-017-4338-6 (PMC5780854; doi:10.1186/s12864-017-4338-6)
Supplement: Supplementary file 4 — The correlation between the term similarity by state-of-art methods and prior knowledge in PubMed. (PDF 134 kb) [file 12864_2017_4338_MOESM4_ESM.pdf]

Additional file 4. The correlation between the term similarity by state-of-art methods and prior knowledge in PubMed. A. The distribution of the similarity score by CroGO method. B. The distribution of the similarity score by VSM method. C. The distribution of the similarity score by ASR method.

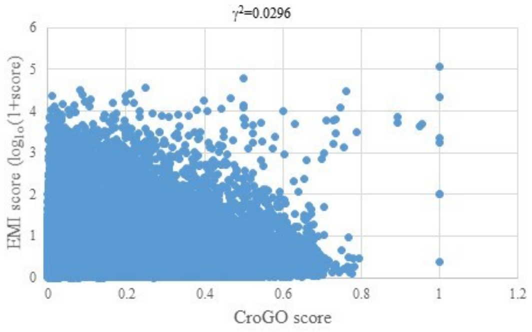

A

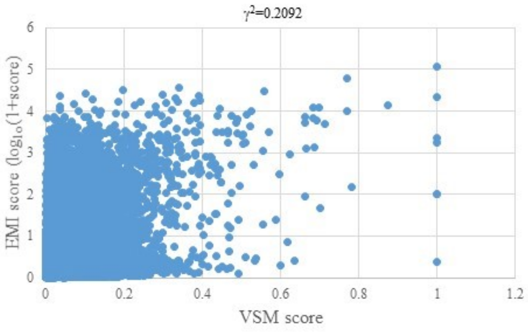

B

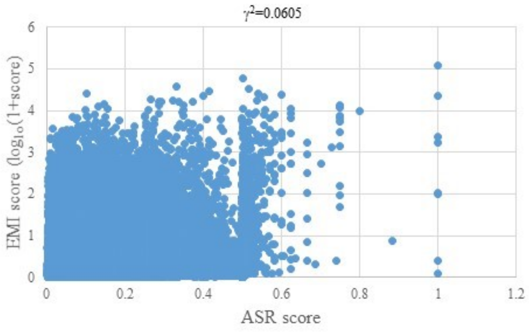

C
