# Supplementary material for: InfAcrOnt: calculating cross-ontology term similarities using information flow by a random walk
Source: BMC Genomics. 2018 Jan 19;19(Suppl 1):919. doi: 10.1186/s12864-017-4338-6 (PMC5780854; doi:10.1186/s12864-017-4338-6)
Supplement: Supplementary file 5 — Disease-related biological process confirmed by literature mining. (PDF 102 kb) [file 12864_2017_4338_MOESM5_ESM.pdf]

Additional file 5. Disease-related biological process confirmed by literature mining.

Biological process	Ranking	References
Diabetes mellitus		
response to oxidative stress	1	(Asmat, et al., 2016)
regulation of protein secretion	2	(Mata, et al., 2004)
regulation of endocytosis	3	(Cester, et al., 1996)
response to reactive oxygen species	4	(Santos, et al., 2013)
negative regulation of apoptotic process	5	(Magee, et al., 2014)
Alzheimer's disease		
positive regulation of apoptotic process	1	(Salemi, et al., 2012)
negative regulation of phosphorylation	2	(Song, et al., 2015)
negative regulation of protein phosphorylation	3	(Song, et al., 2015)
regulation of DNA replication	5	(Yurov, et al., 2011)
Neuroblastoma		
negative regulation of apoptotic process	1	(Li and Nakagawara, 2013)
response to UV	2	(Ramos-Espinosa, et al., 2012)
positive regulation of apoptotic process	4	(Li and Nakagawara, 2013)
cell cycle arrest	5	(Van de Wouwer, et al., 2012)

References

Asmat, U., Abad, K. and Ismail, K. (2016) Diabetes mellitus and oxidative stress-A concise review, *Saudi pharmaceutical journal : SPJ : the official publication of the Saudi Pharmaceutical Society*, **24**, 547-553.

Cester, N., *et al.* (1996) Activation of endothelial cells during insulin-dependent diabetes mellitus: a biochemical and morphological study, *European journal of clinical investigation*, **26**, 569-573.

Li, Y. and Nakagawara, A. (2013) Apoptotic cell death in neuroblastoma, *Cells*, **2**, 432-459.

Magee, T.R., *et al.* (2014) Gestational diabetes mellitus alters apoptotic and inflammatory gene expression of trophobasts from human term placenta, *Journal of diabetes and its complications*, **28**, 448-459.

Mata, A.D., *et al.* (2004) Effects of diabetes mellitus on salivary secretion and its composition in the human, *Molecular and cellular biochemistry*, **261**, 137-142.

Ramos-Espinosa, P., Rojas, E. and Valverde, M. (2012) Differential DNA damage response to UV and hydrogen peroxide depending of differentiation stage in a neuroblastoma model, *Neurotoxicology*, **33**, 1086-1095.

Salemi, M., *et al.* (2012) Three apoptotic genes are upregulated in a patient with Alzheimer's disease and well-differentiated squamous cell carcinoma, *The International journal of biological markers*, **27**, 60-63.

Santos, M.C., *et al.* (2013) Diabetes mellitus increases reactive oxygen species production in the thyroid of male rats, *Endocrinology*, **154**, 1361-1372.

Song, W.J., *et al.* (2015) Enhancement of BACE1 Activity by p25/Cdk5-Mediated Phosphorylation in Alzheimer's Disease, *PLoS One*, **10**, e0136950.

Van de Wouwer, M., *et al.* (2012) Activation of the BRCA1/Chk1/p53/p21(Cip1/Waf1) pathway by nitric oxide and cell cycle arrest in human neuroblastoma NB69 cells, *Nitric oxide : biology and chemistry*, **26**, 182-191.

Yurov, Y.B., Vorsanova, S.G. and Iourov, I.Y. (2011) The DNA replication stress hypothesis of Alzheimer's disease, *TheScientificWorldJournal*, **11**, 2602-2612.
